# Construction of Ultrasonic Tactile Force Feedback Model in Teleoperation Robot System

**DOI:** 10.3390/s21072560

**Published:** 2021-04-06

**Authors:** Yang Liu, Xiaoling Li, Jiarui Lai, Ziming Zheng, Huijin Zhu, Min Li

**Affiliations:** School of Mechanical Engineering, Xi’an Jiaotong University, Xi’an 710000, China; xjtu_liuyang@stu.xjtu.edu.cn (Y.L.); ljr285@stu.xjtu.edu.cn (J.L.); zmzheng121719@stu.xjtu.edu.cn (Z.Z.); zhhjjym@stu.xjtu.edu.cn (H.Z.); min.li@mail.xjtu.edu.cn (M.L.)

**Keywords:** ultrasound array, tactile interaction, force feedback, teleoperation system, regression model

## Abstract

The ultrasonic phased array as an emerging interactive tool is increasingly used for aerial tactile interaction. However, there is almost no method to achieve remote variable force feedback through the ultrasonic phased array as far as we know. This article presents a force tactile feedback method for teleoperating robot systems that tracks the five fingers and forms a focus on the fingertips. First, the perceived size of the focus depends on the input parameters. The influence of the parameters on the physical output pressure intensity was obtained through physical test experiments. Then, the absolute threshold and difference threshold of human perception were studied through psychophysical experimental methods. Finally, the input parameters were selected according to the experimental results. According to the collected data, the construction of the force regression model was completed, and different parameters were mapped to the perceived intensity. The contact force generated in the actual operation is fed back to the haptic system, and the constructed model automatically adjusts the control parameters to ensure that the user’s hand presents a sensory output corresponding to the intensity change. The entire force feedback system is evaluated, and results show that the system shows good perceptual quality.

## 1. Introduction

Robots are increasingly involved in dangerous tasks that are difficult for personnel to perform in complex environments, such as mine clearance, telemedicine, space exploration, industrial operations, etc. [1,2,3,4,5]. Teleoperation is a new type of robot control technology that allows people to avoid on-site dangers and remotely control robots to perform tasks in a safe environment. Immersion and presence are key factors in the robot teleoperation interactive system. The use of tactile force feedback in remote operation can enable the operator to perceive the remote environment in a dynamic interactive manner and improve the operator’s task adjustment ability, thereby enhancing the perception modality [6,7,8]. By using a suitable tactile feedback method, the transparency of the remote operating system can also be improved, and the interactive performance of the system can be improved. The current application of 5G communication technology improves the efficiency of information transmission and greatly reduces system delay. It ensures the stability of dynamic force feedback in teleoperation and provides excellent technical support for the widespread application of teleoperation in the future [9].

Vibration motors are often used in contact tactile interactions, which are convenient, economical, and have significant force feedback effects. Manshad et al. used a single vibration motor to study the role of tactile feedback in mobile payment [10]. Scalera et al. proposed the use of two hand-held joysticks with vibration motors to study tactile stimulation. The combination of vibration motors and other devices brings acceptable tactile perception, but vibration stimulation can only stimulate at a fixed point and cannot reflect the direction of force [11]. González et al. chose the Phantom Omni as the tactile feedback device in the remote operation of industrial robots. It is a small mechanical arm equivalent to a rotary joint. This device can be used in bilateral teleoperation [12]. Unlike point stimulation, such interactive devices can transmit force and torque. The operator perceives force information through contact with the equipment. However, its interaction space is limited, the operator and the device need to be in constant contact, and it is not suitable for simulating the multi-point interaction force transmitted by a multi-finger manipulator. Quek et al. designed a skin deformation tactile feedback device that can provide force information with 3 degrees of freedom [13]. In order to achieve tactile feedback during surgery, the device provides users with force and torque information, simulating normal skin deformation and tangential skin stretching tactile sensation, but its tactile device includes a triangular mechanism and a direct current (DC) motor, which is relatively bulky. Yeh et al. added a piezoelectric actuator to the operating side of the surgical robot to achieve force feedback for remote operation [14]. The piezoelectric actuator has a thin appearance and simple structure and is suitable for haptic feedback in combination with a mechanical structure. In the multi-finger teleoperation robot system, tactile gloves are mainly used for tactile feedback. The three-finger remote control system developed by the German Aerospace Center for the lunar rover prototype and the five-finger grasping system in the virtual reality environment both use the force feedback exoskeleton in the CyberGrasp glove to achieve tactile feedback [15,16,17]. In general, different tactile feedback systems have their own advantages, and different methods are suitable for different application fields and different needs. Vibration motors, mechanical devices, piezoelectric actuators, and haptic gloves are all contact-type tactile interactions. People must contact or wear the device during the interaction. Movement and interaction area in this way will be restricted.

Non-contact tactile interaction breaks the limitations of spatial interaction. Common methods include jet driving force feedback, lasers, and ultrasonic sensor arrays [18,19,20]. Air jets rely on air pulses guided by flexible nozzles to produce a tactile sensation. It is effective in simulating coarse force feedback, but space and time properties are limited. The tactile sensation produced by the laser requires a light-absorbing elastic medium attached to the skin. These two methods are not suitable for multi-finger manipulator force tactile feedback in remote operations.

Ultrasonic phased array formation can form a focal point, and the acoustic radiation pressure generated by it can be effectively sensed by the skin. Compared with other tactile interaction methods, ultrasonic tactile feedback does not require wearing or touching the device. Relevant studies conducted by the University of Tokyo and Johns Hopkins University in Japan have shown that ultrasonic phased arrays have reliable aerial tactile interaction effects [21,22,23]. The ultrasonic phased array can generate multiple movable focal points in space to meet the needs of movement perception and can realize independent force feedback of a single finger. Many laboratories have developed experimental prototypes and semi-commercial products for related research, and researchers have also focused on related human perception and application levels. Sand et al. proposed an aerial tactile feedback system for head-mounted displays, which installed array and hand position sensors on the front surface of the head-mounted virtual reality display [24]. Through the participants completing simple virtual key tapping tasks with or without tactile feedback, the experiment proved that participants had a higher evaluation of self-performance through tactile feedback. Some researchers have also applied ultrasonic tactile feedback in the home environment to simulate the touch perception of a light switch. As far as we know, there is no research on the application of ultrasonic tactile feedback in teleoperation systems.

In the study of a multi-point dynamic force-feedback haptic interaction system, Gonzalo et al. used psychophysical methods in the motor haptic feedback system to study the absolute threshold and the difference threshold. This method can accurately configure the haptic device and create appropriate stimuli to improve the human Machine interactive system [25]. For the five-finger independent force feedback, Yunus et al. developed a new wearable tactile feedback system using a vibration motor. The system can establish a one-to-one mapping between the slave and the master to provide the force perception of a single finger, thereby making the disabled People can recognize the force of each finger [26]. Researchers have proposed a variable motion mapping method by adjusting the motion mapping coefficient to change the feedback stiffness to output accurate force feedback [27]. In the field of ultrasonic haptic feedback, most studies only focus on the impact of rendering parameters on the perceived quality, and no systematic method is proposed for the multi-point dynamic haptic feedback interactive system.

Previous studies mainly focus on the ultrasonic phased array in the field of tactile perception, but there is no research on the changing force feedback of the ultrasonic phased array. This paper proposes a multi-finger changing force tactile feedback method using an ultrasonic phased array during teleoperation. The advantage of this method is that it can approach multi-finger following focus through the ultrasonic sensor array and leap motion. The operator can sense the force transmitted by the manipulator when the arm moves and controls the manipulator. Also, this study develops a force regression model based on control parameters to obtain the feedback and perception of changing force in the interaction process. In the ultrasonic system, the control parameters affecting the output sound pressure intensity are frequency, intensity and focus position. We perform the factor analysis on the parameters through the experiments and develop a force regression model based on the data collected so that the system can adjust the parameters independently to map the changes in the feedback force’s value.

## 2. Description of Teleoperation Robot System

**Figure 1:**
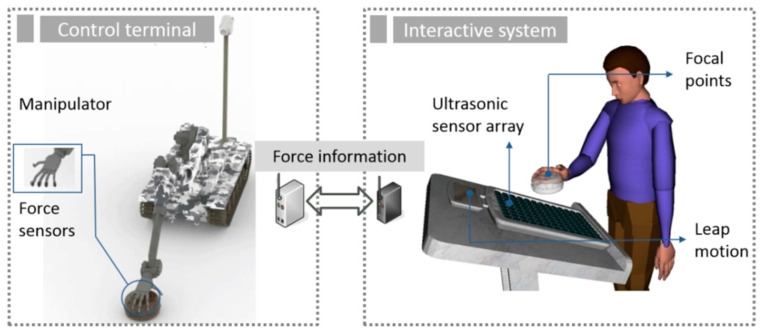
Conceptual diagram of teleoperation robot system.

Figure 1 is a conceptual diagram of the system. When the operator remotely controls the robot to perform operations, the manipulator will perform actions such as grasping the target object and obtain contact force information by in-stalling a pressure sensor on the fingertip of the robot. Dynamic interaction force information can be transmitted back to the human–computer interaction terminal through wireless network transmission. The ultrasonic tactile feedback system we propose as suitable for remote operation is the interactive terminal platform, and the transmitted interactive force information is presented through the ultrasonic array. This allows tracking, recognition and positioning of human fingers by using the leap motion. The ultrasonic phased array emits ultrasonic waves and focuses on the positioning point to realize the tactile perception of human fingertips.

### 2.1. Method of Forming Tactile Focus Points

The ultrasonic phased array can form a focal point in the air through-beam focusing, and the acoustic radiation pressure at the focal point can be sensed by human skin. We use the aerial tactile display obtained from Ultrahaptics for secondary development and related research. We need to achieve real-time, dynamic tactile focus points on five fingers. Therefore, we need to use the phased array to generate five focal points and leap motion for hand tracking. The combination of both means that the focal points generated by the phased array are focused on the thumb, index finger, middle finger, ring finger and little finger. Project programming is mainly designed from two aspects: phased array multi-focus generation and leap motion positioning.

The leap motion acquisition process is mainly divided into the following processes: acquisition of a single frame image, image recognition and analysis, hand model reconstruction, and hand information extraction. For hand information, leap motion can identify information including the position, direction, angle, normal vector, and movement speed of the palm, thumb, index finger, middle finger, ring finger, and little finger. Therefore, it is programmed to realize single-frame image collection finger positioning. For the output of finger information, leap motion expresses the finger model in the form of an array. It recognizes the thumb, index finger, middle finger, ring finger and little finger as the five elements in the array. You can select the element position in the array to determine the information of the finger that you need. It is necessary to determine the position of the fingertip and the direction of the finger to realize the display of the focus on the finger and convert the coordinate system to the coordinate system of the phased array to facilitate subsequent operations.

**Figure 2:**
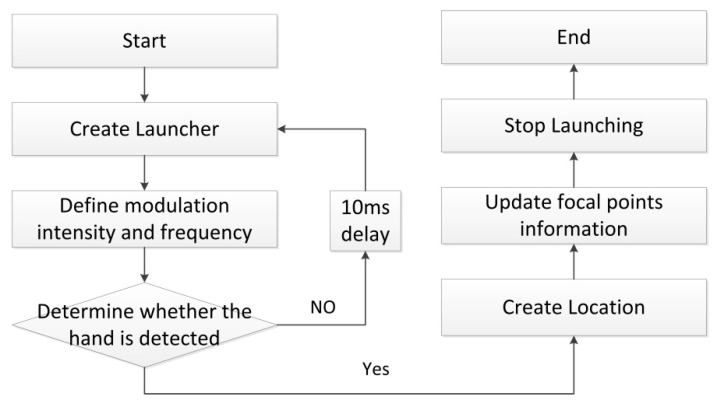
The flow chart of tactile points focusing.

Ultrahaptics’ SDK (Software Development Kit) integrates functions such as focus position, controller launch, modulation, etc., to facilitate design and programming. When transmitting ultrasonic waves into space, amplitude modulation API (Application Programming Interface) that uses sinusoidal signals is used to modulate the ultrasonic waves. When using the AM (amplitude modulation) API, you can continuously send control information, including the modulation frequency, focus position, and focus the intensity of the ultrasound. For the generation of focus, the process mainly includes: creating Ultrahaptics transmitter, defining modulation frequency and intensity, creating control point position, and continuously updating control point information. The process sequence is shown in the Figure 2.

### 2.2. Integration of the System

**Figure 3:**
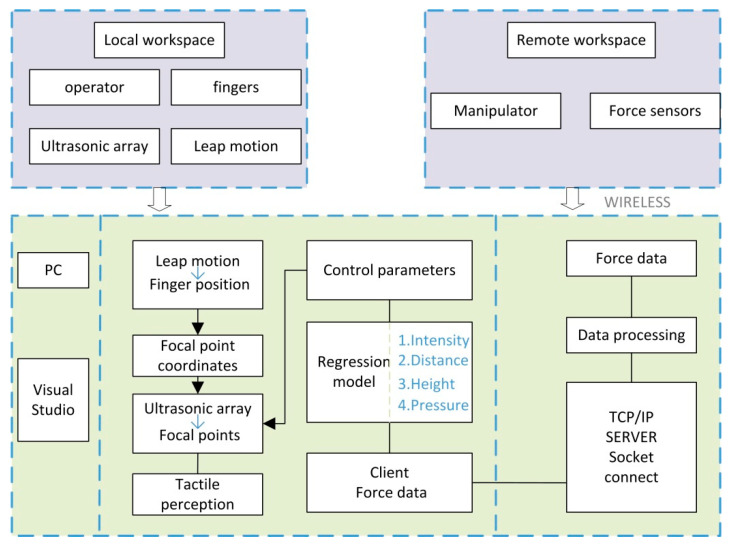
The overall flowchart of our proposed system. It includes the human-computer interaction terminal and the control terminal, as well as the method of data transmission and the method of realizing device control.

The proposed system uses an ultrasonic phased array for dynamic tactile feedback in a robot teleoperation system. The control terminal completes the contact force detection, and the interactive end presents a dynamically changing contact force through an ultrasonic phased array. The PC side completes data processing, transmission and equipment control. The specific technical roadmap is shown in Figure 3. leap motion is needed to determine the focus position, which captures the position of the finger and transmits it to the ultrasonic array control program. The focused pressure is related to the real-time contact force of the control terminal. The pressure information detected by the pressure sensor installed by the manipulator can be transmitted back to the PC by means of wireless network transmission such as an ad hoc network, and the transmitted pressure information needs to be transmitted to the related program that controls the ultrasonic phased array after processing. We realize the transmission of pressure information through network programming. Under normal circumstances, ultrasonic phased array is only used for force sensing. Using the ultrasonic phased array to achieve force feedback requires a new control method. We built a regression model using three parameters: height, distance from centre, and input intensity. The model inputs the received contact force information and can directly output appropriate control parameters to control the ultrasonic array to present a matching tactile focus. The specific construction method of the model will be introduced in detail in the third section.

## 3. Experiment

The ultrasonic phased array can realize tactile perception, but a single control cannot make the ultrasonic phased array output regular or specific tactile power points. In actual use, it can only be used for perception, not force feedback. Therefore, we built a sound field force feedback regression model to realize that the ultrasonic phased array displays a specific magnitude of force feedback at the focal point in the air. The physical force of the focus depends on the input parameters. According to the physical characteristics of the ultrasonic phased array and the related theory of human skin perception of touch, the parameters that affect the perception effect are command intensity, modulation frequency, distance from the centre, and the height of the focus in the working space. Because there are many parameters that affect feedback perception, it is a relatively complicated situation to obtain stable force feedback under different circumstances. Directly constructing the regression model will make the problem complicated and time-consuming. Therefore, we need to complete the factor analysis through experimental methods. Experiment 1 was a physical test experiment. The results of the experiment showed the influence of the aforementioned parameters on physical output pressure. Experiment 2 was a psychophysical experiment to understand the influence of parameters on human perception. Therefore, we obtained the relationship between output pressure and human skin perception. The constructed model mapped different parameters to the perceived amplitude.

### 3.1. Measurement Hardware

**Figure 4:**
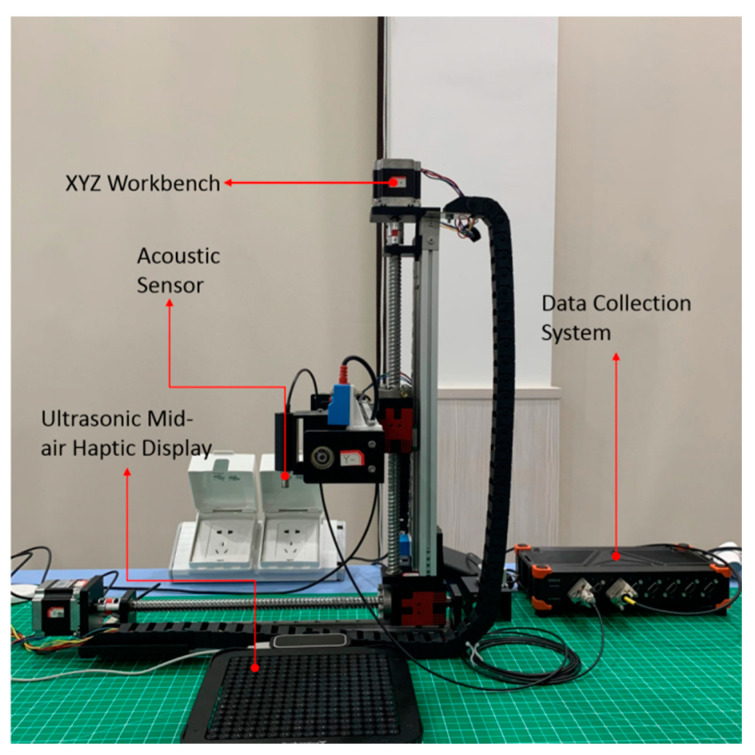
Measurement setup: XYZ workbench, data collector, acoustic sensor, ultrasonic mid-air haptic display.

In order to build a force regression model, we built an experimental platform for testing the sound field to test the characteristics of the sound field under different conditions. An acoustic sensor was used for measurement. Due to the inconvenience of movement when using a balance to measure the force value, only one focus point could be measured at the same time. When the ultrasonic frequency is 40 kHz, the energy loss is only 4%, but if the frequency becomes four times larger, it will lose 50%. At the same time, 40 kHz frequency has a better rendering effect. Therefore, the frequency of the system’s ultrasonic transducer was 40 kHz. The selected acoustic sensor was GRAS 46 BE, which could measure high sound pressure levels at high frequencies. Assembling the XYZ workbench works for precise and systematic spatial measurement with acoustic sensors. The sensors could move freely in three-axis directions. The constructed measurement system is shown in Figure 4. Using the measurement system, we changed the commanding strength, height, and distance from the centre to three parameters, collected five samples for each data point for pressure measurement and moved the sensor to the focus for 5 s to measure each data point. The sample average of each data point was calculated as the final result.

### 3.2. Experiment 1

**Figure 5:**
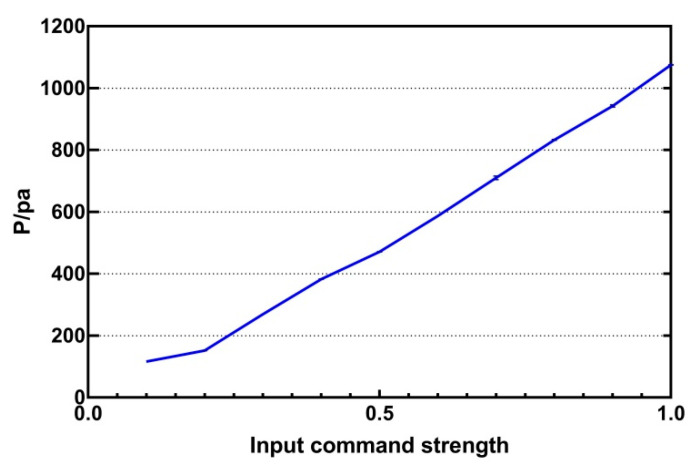
The relationship between input command intensity and output intensity.

Experiment 1 was a physical test experiment. First we discussed the effect of intensity on rendering pressure. In order to observe the changing trend, we fixed the position of the focus. The height was set at 200 mm, the distance from the centre was 0 mm, and the modulation frequency was 240 Hz. The input intensity was 0 to 1, the step length between two consecutive values was 0.1, and the test records ten values. The recorded data are shown in Figure 5. It can be seen that there is a non-linear relationship between intensity and output pressure, but the overall trend shows that the greater the amplitude is, the greater the pressure becomes.

**Figure 6:**
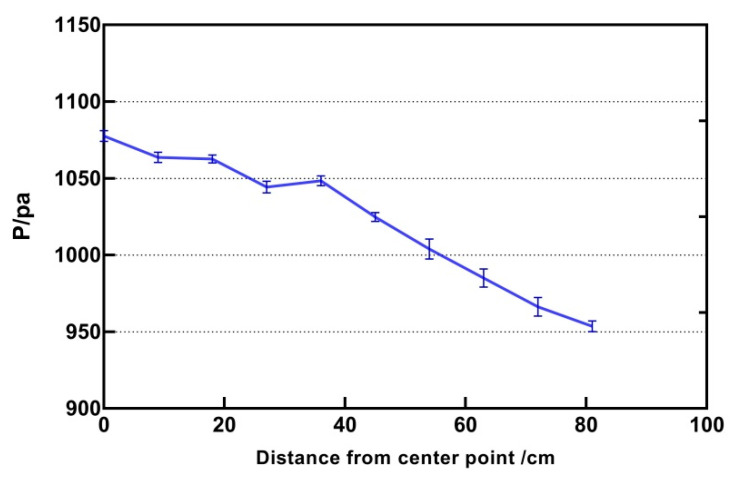
The relationship between the distance from centre point and the output intensity.

In order to judge the influence of the distance to the centre on the intensity of the focus, all other parameters remain unchanged, the frequency was 240 HZ, the height was 200 mm, and the input intensity was 1. The distance from the centre to the target was 9 mm and the maximum distance was 81 mm. The distance was the Euclidean distance from the focal point to the centre. The result is shown in Figure 6. It can be seen that as the focal point moves away from the centre point, the pressure intensity decreases monotonously and presents a non-linear relationship.

**Figure 7:**
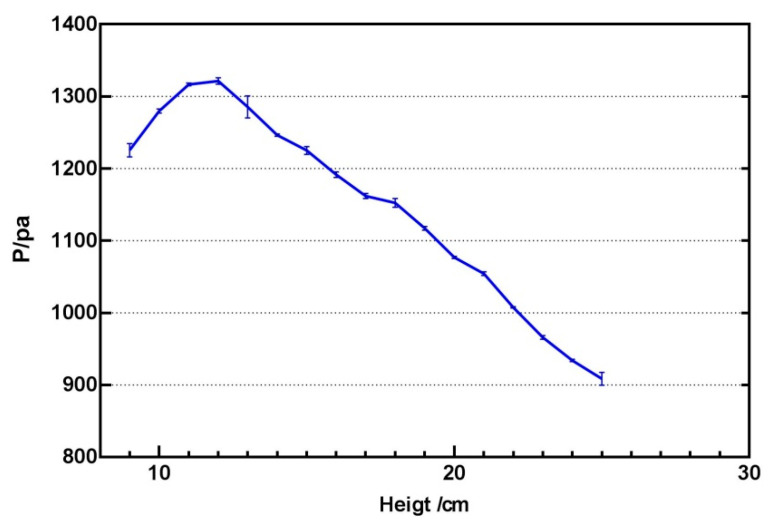
The relationship between height and output intensity.

When studying the influence of the height parameter, a similar experiment was conducted, keeping the frequency at 240 HZ, keeping the distance from the centre at 0 mm, and setting the input intensity to 1. Experiments found that there were many interference points between ultrasonic waves in areas with a height of less than 9 cm, and the perception of the focus with the hand will be greatly interfered with. The spatial perception of heights above 25 cm will be weakened, which is not conducive to tactile perception. The working area should be set between 9–25 cm. It is observed from experimental Figure 7 that the relationship between the two is non-linear, and the output pressure intensity will decrease as the height increases.

### 3.3. Experiment 2

Experiment 2 was a psychophysical experiment. To understand the tactile perception under ultrasound stimulation, we observed the relationship between physical stimuli and user responses. Our experiment mainly determines two values, namely the absolute threshold and the differential threshold. The absolute threshold is the minimum intensity at which the stimulus can be detected, and the differential threshold is the smallest change where the subject can perceive the increase or decrease of the intensity of the stimulus. The experimental methods used to estimate the sensory threshold can usually use psychophysical experimental methods. We chose the adaptive ladder method for evaluation to provide accurate results in less time.

#### 3.3.1. Participants

Ten participants (3 females and 7 males, aged between 23–30) were recruited into the experiment. They were all right-handed and all subjects had no disability and no physical injury affecting palm sensitivity.

#### 3.3.2. Procedure

During the experiment, a piece of foam plastic was placed above the array in a fixed relative position. During the experiment, participants put their palms on the foam with their palms facing down to ensure that the palms were in a fixed position relative to the transducer array and that the palms were as horizontal as possible. The position of the ultrasonic focus was set 20 cm above the upper surface of the array. During the experiment, the ultrasonic transducer array will emit audible sounds. When the amplitude is different, the volume of the sound is also different. We asked participants to wear headphones when listening to the pre-recorded noise of the transducer array and natural rain so that they could not hear the changing sound of the transducer array.

We conducted experiments by adaptive ladder program with variable step length “1up-2down”. The experiment was divided into stair case A and stair case B. At the beginning of the experiment, in experiment A, the subjects were provided with higher intensity stimulation, and in experiment B, the subjects were provided with lower stimulation. The duration of the stimulus was about 3 s. The participant was asked if they feel the stimulus. If the participant answered yes twice, the stimulus level would be reduced by one level. If the participant answers no, the stimulus level would be increased in the next test. After each reversal, the stimulus step length was reduced to ensure a more accurate adjustment. The experiment was stopped after eight reversals, and the average value of the stimulus level at the point of the last four reversals was the absolute threshold. The step size is 0.4, 0.3, 0.2, 0.1, 0.05, 0.03 and 0.01.

In the differential threshold experiment, participants were required to compare the two stimulus intensities and answered whether they feel the difference between them. Each test included the first stimulus reference (SR) and comparative stimulus (CS). Each test stayed for 3 s. In the first experiment, the two stimuli were chosen to have larger differences. Participants were asked whether they felt the difference, and the participants answered yes that the stimulation level would decrease in the next stage, and vice versa, the stimulation level would increase. Every time the response result changed, we made the stimulus step smaller to make the adjustment more convenient and accurate. The steps used are 0.5, 0.4, 0.3, 0.2, 0.1, 0.05, 0.03, and 0.02. Similarly, after eight reversals, the differential threshold is calculated based on the average of the last four reversal points.

#### 3.3.3. Results

**Figure 8:**
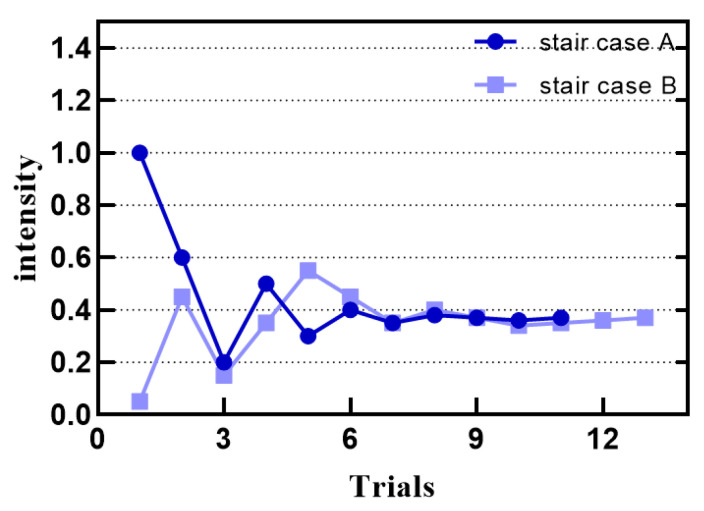
The result of the absolute threshold measurement. An example: absolute threshold measurement result of two staircase programs.

Figure 8 is an example result of the experiment. The experimental results show that when the input intensity value is 0.39, it is the smallest perceived intensity.

**Figure 9:**
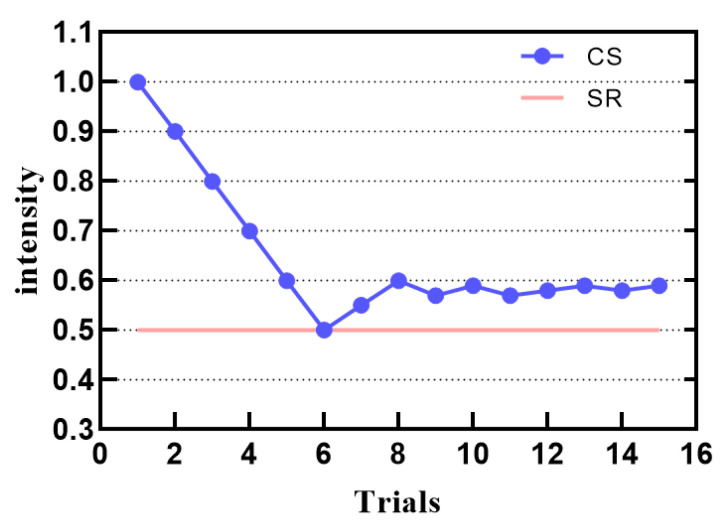
Psychophysical adaptive staircase for subject 2 with a reference flow rate of 0.5.

Figure 9 is an example of a differential threshold experiment. When the input intensity value interval is 0.1, there is the smallest perceptual change.

### 3.4. Regression Model

In the previous test experiments and perception experiments, we learned that the input command strength, height, distance from the centre, and modulation frequency will all affect the subsequent force feedback perception. Our goal is that the system can automatically output the output force corresponding to the change of the input force so that the operator can obtain a stable perception. According to the threshold detection experiment, we can know that under the continuous change of the control intensity, the change of the tactile feedback force generated by the ultrasonic phased array can be perceived by the human finger. The parameter values in all cases are considered, and a regression model is constructed for the mapping between parameters and output. This is the most direct realization method. But this method has some problems. First, we perform a factor analysis. The first is the frequency. Previous studies have shown that when different modulation frequencies are used, the perception of human skin is different and exhibits non-linear changes. As the frequency changes, the measured physical force will not change accordingly. We cannot directly map frequency to output force. The changes in the three parameters of command strength, height, and distance from the centre will not only lead to changes in perception but also changes in physical force, and the direction of change is the same. The different perceptual results of changing the frequency and the perceptual results of changing the commanded intensity will largely overlap. Through analysis, we do not need to set the frequency as the parameter of the regression model. In order to present the maximum tactile perception, we set the modulation frequency of the system to the human body’s best perception frequency, 240 Hz. Therefore, our input parameters are set as the input command strength, height, and distance from the centre.

**Figure 10:**
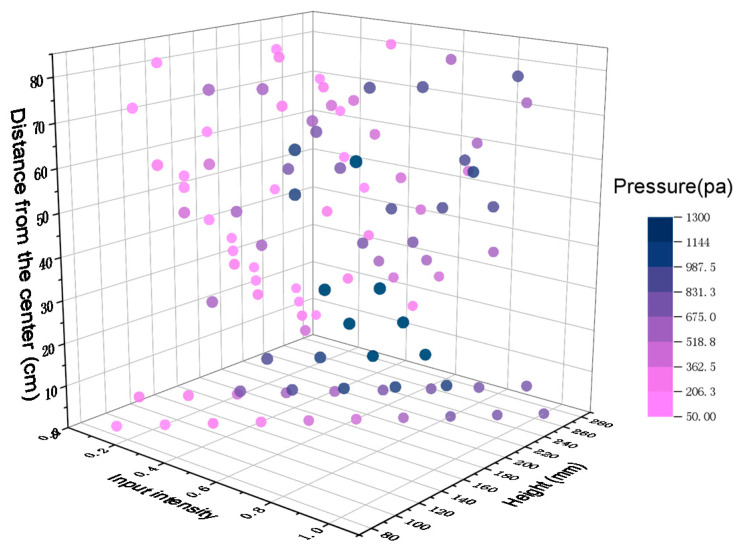
Three-dimensional display of test data points. The *x*-axis is the intensity of the input command, the *y*-axis is the height, the *z*-axis is the distance from the centre point, the test points are characteristic data points of different parameter combinations, and the measured pressure is indicated by color.

According to our experience when the ultrasonic phased array is arranged in a square, our actual test data do not need to cover the entire space, and we select typical representative points in the horizontal and vertical directions for testing. The tested data points are shown in Figure 10. The three coordinate axes are the input command intensity, height, and distance from the centre. The pressure at each data point is indicated by colour. The test data set is used for multiple regression. Formula 1 represents the regression model, where P is the output sound pressure value, a is the input command intensity, z is the height, and d is the distance from the centre.

P = 98.136 + 840.889 ∗ a − 0.548 ∗ z + 13.588 ∗ d + 266.198 ∗ a^2^ − 0.003 ∗ z^2^ − 0.290 ∗ d^2^ (1)

**Figure 11:**
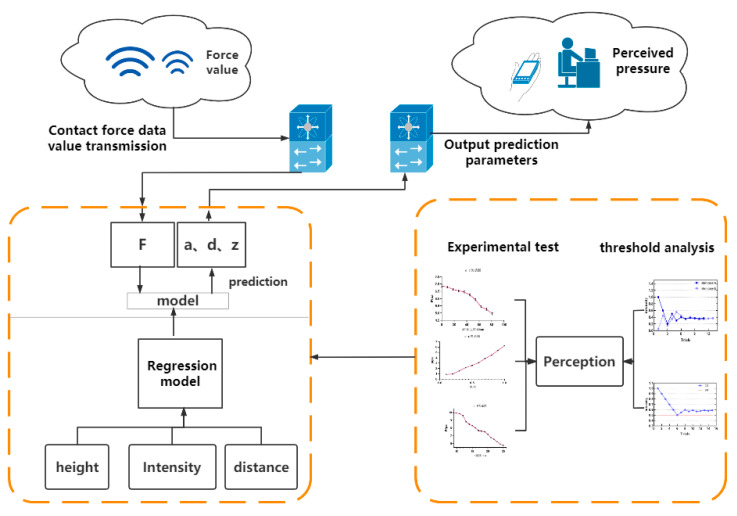
Frame diagram of real-time force feedback system.

The entire sound field force feedback system is shown in Figure 11. The robot end inputs the force information and position information of the interaction end into the force feedback system and then constructs a good regression model to output the predicted value to the ultrasonic tactile feedback system to control the ultrasonic tactile feedback system. The ultrasonic tactile feedback system provides a specific magnitude of force feedback corresponding to the input force for the operator’s perception.

## 4. Results and Discussion

### 4.1. Measurement

**Figure 12:**
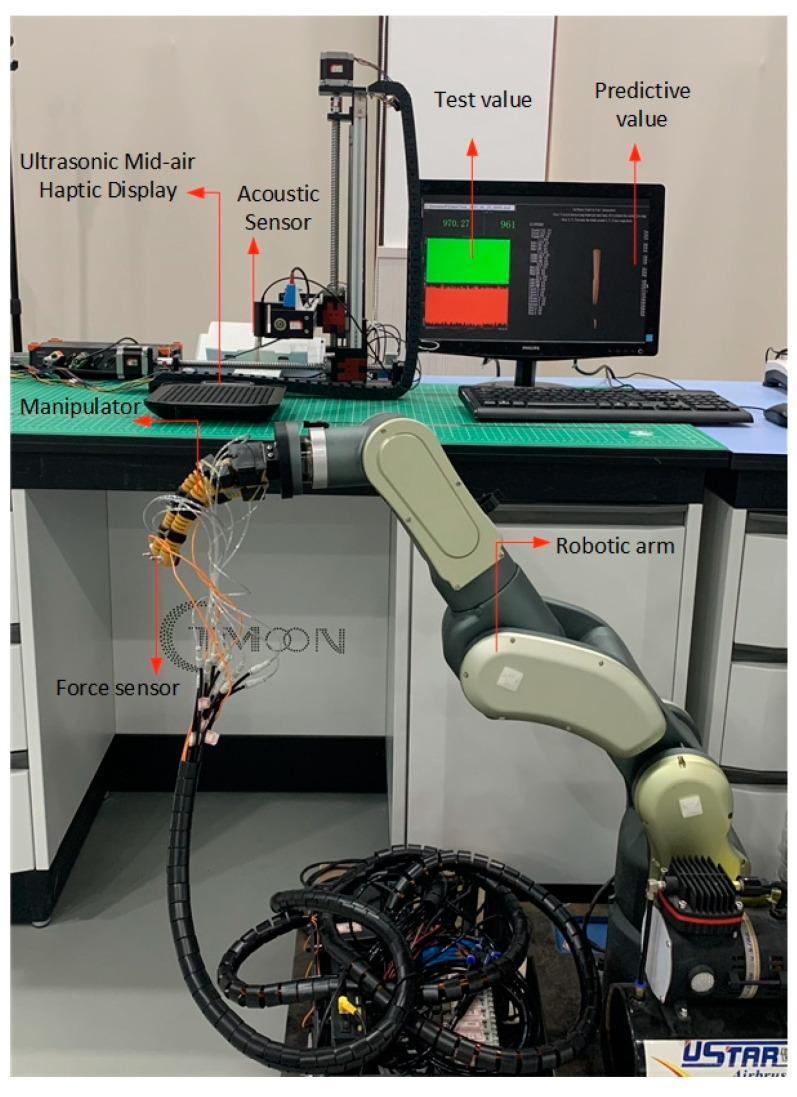
Test and verification experiment diagram in teleoperation system.

A physical test experiment was designed to check the accuracy of the output pressure after using the regression model. As shown in Figure 12, the interactive part was an ultrasonic phased array combined with leap motion. The sound pressure at the ultrasound focus point was tested using an acoustic sensor GRAS 46 BE (GRAS Sound & Vibration, Holte, Denmark). The 8-channel multifunctional data acquisition module SIRIUS (DEWESoft, Kumberg, Austria) and DEWESoft X (DEWESoft, Kumberg, Austria) were selected for data collection and analysis. A lightweight robotic arm (RobotAnno, Guangzhou, China) was used as a remote control terminal. A pneumatic bionic hand was installed at the end of the robotic arm, and the bionic hand controls the fingers to perform gripping actions through the pipes connected to an air pump. The pressure data of the contact between the finger and the object were measured by the pressure sensor BSLM-2 (BUFSON, Shijiazhuang, China) on the fingers of the bionic hand.

In the experiment, the collected pressure data was directly transmitted back to the PC through wireless ad-hoc network communication. After processing the pressure data into a numerical value corresponding to the sound pressure, this numerical value was used as the predicted value of the experiment. The predicted value was input to the regression model, and then the control parameters were output so that the ultrasonic phased array generated the corresponding focus point at the position where the leap motion detects the fingers. The intensity value of the gathering point was used as the test value in the experiment. Two sets of test experiments were set up. The first group was given a unique predicted value, and the test value was compared with the predicted value at different points on the horizontal plane. The second group set different predicted values at points of different heights and compared the test value with the predicted value. In addition, repeated-measures analyses of variance (ANOVA) were performed to compare the data. All data were assessed for the approximation to a normal distribution and sphericity, and when necessary, degrees of freedom were adjusted using the Greenhouse-Geisser adjustment. All data were performed using SPSS statistical software (V22, Chicago, IL, USA). When the test result showed significance of difference, it was labeled as “*p* < 0.05” or “*p* < 0.01”, otherwise, “*p* > 0.05” was labeled.

**Figure 13:**
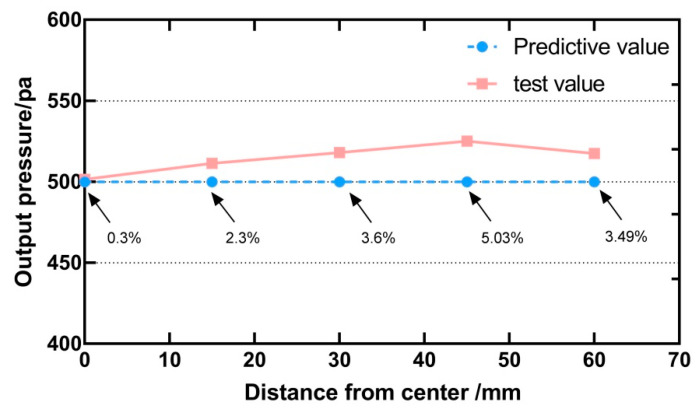
The predicted and measured values at different positions from the centre point on the horizontal plane. The value indicated by the arrow is the relative error.

The height of the selected points in the first group was 250 mm, and the distance from the centre was different. The required output power was set to 500 pa, which was the predicted value. The pressure value generated by the ultrasonic array using the regression model was the test value. Five data points were tested, the reading started from the centre, with a step length of 15 mm, and the experimental results are shown in Figure 13. There is a small error between the predicted value and the test value. The errors of the five points are 0.3%, 2.3%, 3.6%, 5.03%, and 3.49% respectively. The average error is 2.94%. Result of the repeated measures ANOVA shows that there were significant differences between the predicted value and the test value [F (1, 4) = 13.88, *p* < 0.05].

**Figure 14:**
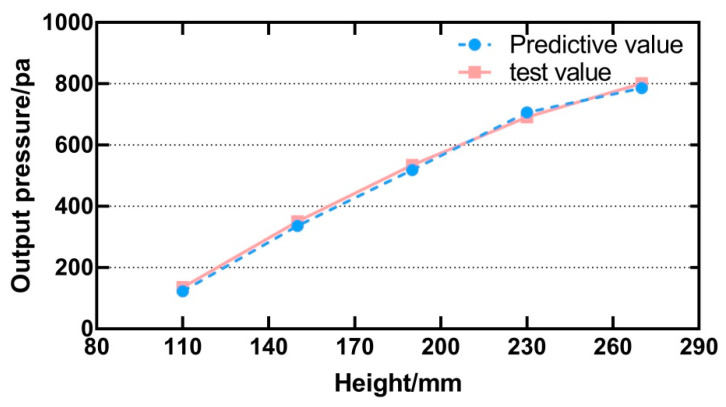
Test values and predicted values at different heights on the vertical line where the centre point of the array is located.

The second group selected the point at the centre. The height started from 110 mm, the step length was 30 mm, five data points were tested, and the predicted value randomly inputted from small to large. The experimental result is shown in Figure 14. The test value is very close to the predicted value, and there is only a slight error. Result of the repeated measures ANOVA shows that there is no significant difference between the predicted value and the test value [F (1, 4) = 2.10, *p* > 0.05]. Experiments show that the output of the ultrasonic array is controlled well by the regression model.

### 4.2. Discussion

As mentioned earlier, the tactile points focused by the ultrasonic array were generally used for tactile perception in different situations. The output pressure of the equipment was not usually concerned about this, and changed in an irregular form in the process of movement perception. However, in some cases, we need to perceive changes in contact force through touch. For example, in the robot teleoperation system, the contact points generated by the ultrasonic array were used to express the change of the contact force during the robot’s operation, and the required tactile interaction could be obtained more realistically and naturally. Therefore, the force regression model could change the control parameters in real time and output the tactile focus with a specific pressure level. It can be seen from Figure 14 that the ultrasonic array can render more accurate tactile points of a specific size. Although there was a difference between the test value and the predicted value at different points on the horizontal plane, according to human perception characteristics, this degree of error will not cause a significant difference in perception [28,29].

In this study, the method set the best perception frequency to avoid complicated combinations. Therefore, the input command intensity needed to be adjusted in different positions. At the farthest edge point of the array, the maximum output force was when the input command intensity value was the maximum. Due to the characteristics of the ultrasonic array to achieve tactile perception, it may happen that when the contact force was large, the focused ultrasonic pressure cannot be equal to the contact force. The overall output pressure could be scaled down according to needs and effects. In this case, the size of the array became the limiting factor for the size of the focus output pressure, and the expansion of the array could be considered to meet the system requirements.

## 5. Conclusions

This paper presents a method of using the ultrasonic phased array for non-contact tactile interaction in the teleoperation system. The method enables the ultrasonic phased array to input changing force tactile feedback while following the movement of the finger to focus. The main conclusions were summarized as below:

1. Leap motion was used to complete the position of the finger during the positioning operation. The ultrasonic phased array focused on adjustable tactile points, which were used to feed back the contact force between the manipulator and the object.
2. We conducted the physical test experiments and psychological physical experiments to investigate the influence of different parameters on the output intensity of the constructed focus and then completed the factor analysis.
3. The modulation frequency was set as the human body’s best perception frequency, and the three parameters, including height, distance from the centre, and input command strength, were set as system control parameters. The regression model was constructed according to the collected characteristic data points, and the input parameters were mapped to the output intensity.
4. A physical test experiment was designed to check the accuracy of the output pressure after using the regression model. The results from repeated measures ANOVA showed that there is a significant difference between the test value and the predicted value at different points on the horizontal plane, but the average error is only 2.94%, which will not cause a significant difference in perception. There is no significant difference in test results at different heights.

## Data Availability

The data presented in this study are available on request from the corresponding author.
